# Comparison of darbepoetin alfa dosed weekly (QW) vs. extended dosing schedule (EDS) in the treatment of anemia in patients receiving multicycle chemotherapy in a randomized, phase 2, open-label trial

**DOI:** 10.1186/1471-2407-10-581

**Published:** 2010-10-25

**Authors:** Lee Schwartzberg, Ronald Burkes, Barry Mirtsching, Timothy Rearden, Peter Silberstein, Lorrin Yee, Amy Inamoto, Tom Lillie

**Affiliations:** 1 West Clinic, Memphis, TN, USA; 2 Mount Sinai Hospital, Toronto, Ontario, Canada; 3 Center for Oncology Research and Treatment, Dallas, TX, USA; 4 Hematology Oncology Consultants Inc, St Louis, MO, USA; 5 Creighton University School of Medicine, Omaha, NE, USA; 6 Northwest Medical Specialties, PLLC, Tacoma, WA, USA; 7 Amgen Inc., Thousand Oaks, CA, USA

## Abstract

**Background:**

Chemotherapy-induced anemia (CIA) is responsive to treatment with erythropoiesis-stimulating agents (ESAs) such as darbepoetin alfa. Administration of ESAs on a synchronous schedule with chemotherapy administration could benefit patients by reducing clinic visits and potentially enhancing on-time chemotherapy delivery.

**Methods:**

This phase 2, 25-week, open-label study evaluated the noninferiority of darbepoetin alfa administered weekly vs. as an extended dosing schedule (every 2 or 3 weeks) in patients with CIA. Patients were randomized 1:1 to an extended dosing schedule (EDS: darbepoetin alfa 300 μg Q2W if chemotherapy was QW, Q2W, or Q4W or darbepoetin alfa 500 μg Q3W if chemotherapy was Q3W) or weekly (150 μg QW regardless of chemotherapy schedule). Stratification factors included chemotherapy cycle length, screening hemoglobin (<10 g/dL vs. ≥10 g/dL), and tumor type (lung/gynecological vs. other nonmyeloid malignancies). The primary endpoint was change in hemoglobin from baseline to Week 13.

**Results:**

Seven hundred fifty-two patients (374 QW patients; 378 EDS patients) received ≥1 dose of darbepoetin alfa and were included in the analysis. Demographics and disease state were similar between groups. Seventy-one percent of patients in the EDS group and 76% in the QW group achieved the target hemoglobin of ≥11.0 g/dL. There was a minimal difference in the primary endpoint of mean change in hemoglobin (baseline to Week 13) between the QW and the EDS groups (-0.04 g/dL; 95% confidence interval: -0.26, 0.17 g/dL). The upper limit of the 95% confidence interval was less than the prespecified limit of <0.75 g/dL, supporting noninferiority of the EDS dosing schedule. Reported adverse events were similar between groups. A slight increase in transfusions was reported in the QW group.

**Conclusion:**

Darbepoetin alfa, when administered synchronously with chemotherapy, on an EDS appears to be similarly efficacious to darbepoetin alfa weekly dosing with no unexpected adverse events. This study provides prospective data on how multiple dosing regimens available with darbepoetin alfa can be synchronized with chemotherapy administered across a range of dosing schedules.

**Trial registration:**

ClinicalTrials.gov Identifier NCT00144131.

## Background

Chemotherapy-induced anemia (CIA) is a common complication in patients receiving myelosuppressive chemotherapy, contributing to fatigue and reduced quality of life [1]. Darbepoetin alfa is a recombinant human erythropoietin approved for treating the symptoms of CIA by increasing hemoglobin concentrations and thereby decreasing the incidence of red blood cell (RBC) transfusions [2-5]. The goal of erythropoietin treatment in the CIA setting is to maintain hemoglobin at a level to avoid transfusion [6].

In the US, the current darbepoetin alfa prescribing information includes two dosing regimens for the treatment of CIA [6]. The original approval for CIA in 2002 stipulated a 2.25 μg/kg weekly (QW) dosing regimen. A subsequent amendment added an extended dosing schedule (EDS) of 500 μg every three weeks (Q3W) to the prescribing information in 2006. In the EU, a weight-based dosing of 6.75 μg/kg Q3W is approved for treatment of CIA. Nonetheless, two US medication-use evaluation studies, utilizing retrospective chart analyses, have shown that an unindicated initial dose of 200 μg every two weeks (Q2W) was the most common dosing choice by physicians at hospital and community oncology centers in these two studies [7,8]. This alternate extended dosing regimen most likely arose because many common chemotherapy regimens progress on a similar Q2W schedule; thus this darbepoetin alfa regimen of 200 μg Q2W may provide greater patient convenience and enhance adherence to Q2W chemotherapy regimens by reducing the number of patient visits.

The primary objective of this study was to compare the efficacy (noninferiority) with respect to change in hemoglobin of extended dosing darbepoetin alfa (EDS: Q3W or Q2W) vs. QW darbepoetin alfa for treatment of anemia in patients with nonmyeloid malignancies receiving multicycle chemotherapy. In addition, secondary objectives evaluated the impact of darbepoetin alfa EDS vs. QW administration on patient-reported outcomes, resource utilization, and safety parameters.

## Methods

### Study population

Eligible patients were at least 18 years of age with active nonmyeloid malignancies (including lymphocytic leukemias) and anemia defined as screening hemoglobin of less than 11.0 g/dL. Additionally, eligible patients were required to be receiving chemotherapy with a planned additional eight weeks of cytotoxic chemotherapy. Patients were also required to have an Eastern Cooperative Oncology Group (ECOG) performance status of 0 to 2. Patients with acute myelogenous leukemia, chronic myelogenous leukemia, or myelodysplastic syndromes were ineligible, as were patients with angina, hypertension, history of pure red cell aplasia, or any RBC transfusion within 28 days of screening. Patients who had received or were planning to receive myeloablative radiation therapy or who had received a bone marrow or stem cell transplant six months prior to screening were also ineligible, as were patients who had received any erythropoiesis-stimulating agent (ESA) within 28 days prior to screening.

### Study drug

Darbepoetin alfa (Aranesp^®^, Amgen Inc., Thousand Oaks, California) was provided as a clear, colorless, sterile, preservative-free protein solution containing 100 μg, 150 μg, 200 μg, 300 μg, or 500 μg of darbepoetin alfa per mL.

### Study design

**Figure 1:**
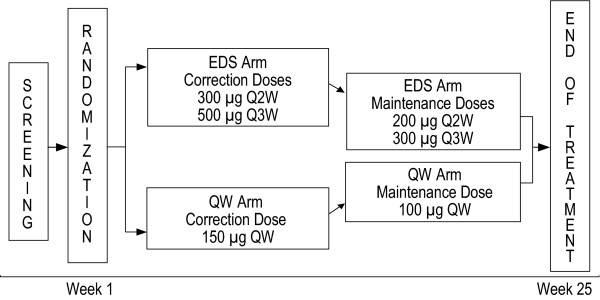
**Study design**. Stratification was by chemotherapy cycle length (≤35% QW), hemoglobin at screening (<10 g/dL vs. ≥10 g/dL), and tumor type (lung/gynecological vs. other nonmyeloid malignancies).

This was a 25-week, randomized, controlled, open-label, multicenter clinical trial. The study design is summarized in Figure 1. Eligible patients were randomized 1:1 to EDS (darbepoetin alfa 500 μg Q3W if chemotherapy was administered Q3W or darbepoetin alfa 300 μg Q2W if chemotherapy was administered QW, Q2W, or Q4W) vs. QW (darbepoetin alfa 150 μg QW regardless of chemotherapy schedule) arms using an interactive voice-response system. Randomization was stratified by chemotherapy cycle length (with no more than 35% of patients receiving QW chemotherapy), hemoglobin at screening (<10 g/dL vs. ≥10 g/dL), and tumor type (lung/gynecological vs. all other nonmyeloid malignancies). ESA efficacy may vary with type of chemotherapy, such as platinum-based therapies, and certain tumor types (such as lung) are more likely to be treated with platinum-based chemotherapy. We therefore stratified by tumor type to minimize the role that chemotherapy agent could have on outcomes.

In the EDS group, patients received darbepoetin alfa 300 μg Q2W or 500 μg Q3W (300 μg Q2W if chemotherapy was administered QW, Q2W, or Q4W; 500 μg Q3W if chemotherapy was administered Q3W). In the QW group, patients received 150 μg darbepoetin alfa weekly regardless of their chemotherapy treatment schedule. Patients who achieved the target hemoglobin of ≥11.0 g/dL or who had a rapid increase in hemoglobin (>1.5 g/dL in three weeks for patients receiving darbepoetin alfa Q3W or >2.0 g/dL in four weeks for patients in the QW or Q2W group) experienced dose reductions. Patients who exceeded the hemoglobin threshold of 13.0 g/dL had darbepoetin alfa withheld until hemoglobin was ≤12.0 g/dL.

The primary endpoint for the study was the change in hemoglobin from baseline to Week 13. Secondary endpoints included transfusions from baseline to Week 13, improvement in Functional Assessment of Cancer Therapy-Fatigue (FACT-F) scores, and the frequency of clinical procedures and impact of clinic visits on activities of daily life. Tertiary endpoints included hematopoietic response from baseline to Week 13 and from baseline to end of treatment, and also the average hemoglobin concentration maintained after the target hemoglobin level was achieved. End of treatment was defined as Week 25, but could be as early as Week 17 if a patient had at least 8 weeks of chemotherapy and an additional 8 weeks on study following the last dose of chemotherapy.

This study was conducted at 130 sites in the US and Canada. The study was conducted in accordance with the Declaration of Helsinki and the principles of good clinical practice. All patients gave their written consent for study participation. Study approval was obtained from each site's Institutional Review Board or Institutional Ethics Committee. The study ran from June 2005 to September 2006.

### Statistical analysis

In order to establish the noninferiority margin for the primary endpoint, the results of two large, phase 3, placebo-controlled trials of 12 weeks duration were examined: one in patients with lung cancer (n = 314) [5], and one in patients with lymphoproliferative disease (n = 344) [3]. In a combined analysis of the two trials, the difference between the darbepoetin alfa (2.25 μg/kg QW) and placebo groups with respect to the mean (95% confidence interval [CI]) change in hemoglobin from baseline to Week 13 was 1.5 g/dL (1.2, 1.8 g/dL). The noninferiority margin for change in hemoglobin used in this study, 0.75 g/dL, was based on the concept of preserving 50% of the difference in the mean change in hemoglobin from baseline to week 13 in the active arm of the placebo-controlled trials [3,5]. If the upper limit of the 95% CI for the difference in the mean change in hemoglobin between the QW regimen of darbepoetin alfa and the EDS regimen of darbepoetin alfa (QW - EDS) was not more than 0.75 g/dL, then the conclusion from this study would be that EDS of darbepoetin alfa is noninferior to darbepoetin alfa administered QW.

Assuming that the difference in hemoglobin between the 2 treatment groups is 0.1 g/dL and that the standard deviation (SD) for the difference in the mean change in hemoglobin from baseline to Week 13 between the 2 treatment groups was 2.6 g/dL, a total of 750 randomized patients (375 patients per treatment group) would provide 92% power to demonstrate noninferiority of EDS to QW dosing schedule based on a noninferiority margin of 0.75 g/dL and a Type I error rate of 5%.

All efficacy analyses were prespecified to be analyzed on patients who were randomized and received at least 1 dose of study drug. Both last-value-carried-forward (LVCF) imputation and available-data approaches were used for the primary endpoint (change in hemoglobin).

An analysis of variance (ANOVA) model was used to evaluate the change in hemoglobin from baseline to Week 13. The model contained treatment group, chemotherapy cycle length (QW vs. Q2W vs. Q3W), screening hemoglobin (<10 g/dL vs. ≥10 g/dL), and tumor type (lung/gynecological vs. other nonmyeloid malignancies) as explanatory variables and change in hemoglobin from baseline to Week 13 as the response variable. For dichotomous efficacy endpoints (receiving a transfusion from baseline through Week 13, achieving the target hemoglobin level, and achieving a hematopoietic response), the proportion of patients in each treatment group having the event was estimated by the Kaplan-Meier (KM) method; 95% CIs for these parameters were constructed using Greenwood's formula for the variance and normal approximation of the KM proportion. Mean change in the FACT-F subscore from baseline to Week 13 and from baseline to end of treatment was analyzed using an ANOVA model, and the 2-sided 95% CI for differences between groups was determined. A ≥3-point increase in FACT-F score was deemed to be clinically significant [9].

The frequency of clinical procedures performed and frequency of activities impacted by clinic visits from baseline to Week 13 and from baseline to end of treatment was collected for each treatment group.

### Safety

Adverse events were categorized by system organ class and preferred term according to the MedDRA (v 9.0) dictionary.

## Results

### Patient demographics and characteristics

**Figure 2:**
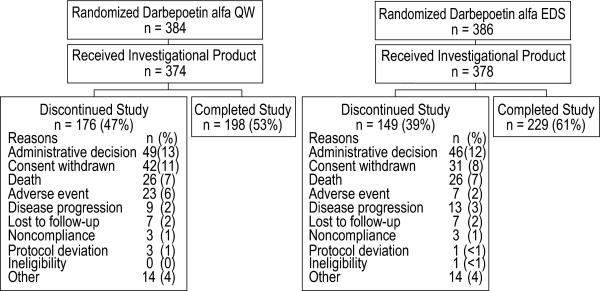
**Patient disposition (CONSORT diagram)**. Reasons for study discontinuation were similar between treatment groups.

A total of 770 patients were enrolled from 130 sites, including 386 patients randomized to EDS dosing and 384 patients randomized to QW dosing (Figure 2). Among those randomized, 378 patients received darbepoetin alfa in the EDS arm, and 374 were dosed in the QW arm. Of patients randomized but not receiving study drug (8 for EDS, 10 for QW), the most common reasons for not receiving study drug were that the patient either delayed or did not have chemotherapy (5 patients) or withdrew consent (4 patients), with a variety of reasons for the remaining 9 patients (such as screening failure, patient was hospitalized, patient was on a different chemotherapy schedule, etc.).

Demographic characteristics were mostly balanced between groups (Table 1). Women outnumbered men in this study, comprising roughly two-thirds of the sample. Nearly half of patients in both groups were age 65 years or older. Breast, lung, and gastrointestinal tumors were the most common primary tumor types. Overall, about half the patients had stage IV disease. Among patients with small cell lung cancer, approximately half were diagnosed with extensive disease. Mean baseline serum erythropoietin levels were slightly lower in the EDS group than the QW group (73.4 mU/mL and 86.9 mU/mL, respectively).

**Table 1:** Patient demographics and clinical characteristics

	QW	EDS
	(n = 374)	(n = 378)
Sex, n (%)		
Men	148 (40)	126 (33)
Women	226 (60)	252 (67)
Race, n (%)		
White	301 (80)	309 (82)
Black or African American	41 (11)	41 (11)
Hispanic or Latino	21 (6)	12 (3)
Asian	5 (1)	7 (2)
Japanese	2 (1)	1 (<1)
Other	4 (1)	8 (2)
Mean (SD) age, years	62.4 (12.5)	62.8 (13.0)
95% CI	61.2, 63.7	61.5, 64.2
Age ≥65 years, n (%)	177 (47)	180 (48)
Age ≥75 years, n (%)	68 (18)	80 (21)
Primary tumor type, n (%)		
Breast	100 (27)	112 (30)
Lung	91 (24)	101 (27)
Gastrointestinal	89 (24)	72 (19)
Gynecologic	29 (8)	30 (8)
Leukemia	26 (7)	22 (6)
Genitourinary	17 (5)	20 (5)
Other^a^	22 (6)	21 (6)
Patients with stage IV cancer, n (%)^b^	183 (52)	179 (50)
Mean (SD) baseline Hb^c ^(g/dL)	10.1 (0.9)	10.1 (0.8)

^a^May include head and neck and hematologic cancer

^b^Excludes patients with small cell lung cancer

^c^Baseline Hb values within 28 days after RBC transfusion were not excluded or imputed

QW = once weekly; EDS = extended dosing schedule; SD = standard deviation; Hb = hemoglobin

Patients in both groups received similar average weekly doses of darbepoetin alfa. The average weekly dose administered to the EDS group (106.8 μg) was comparable to the average weekly dose administered to the QW group (98.2 μg). The mean weight-adjusted weekly dose administered to the EDS patients (1.5 μg/kg/dose) was similar to the QW patients' dose (1.4 μg/kg/dose).

Two hundred twenty-nine patients (61%) in the EDS arm and 198 patients (53%) in the QW arm completed the study (Figure 2). Three hundred twenty-five patients did not complete the study (149 EDS patients [39%] and 176 QW patients [47%]). Common reasons for study discontinuation included administrative decision, consent withdrawn, death, and adverse event (Figure 2).

### Efficacy endpoints

The primary efficacy endpoint was the change in hemoglobin from baseline to Week 13. Data were adjusted by screening hemoglobin category, chemotherapy cycle length, and tumor type. Employing the LVCF approach, a minimal difference was observed between the QW group and EDS groups in the least squares mean [95% CI] change in hemoglobin from baseline to Week 13 (0.90 g/dL [0.74, 1.07] and 0.95 g/dL [0.79, 1.11], respectively) (Table 2). When the available-data approach was used, the least squares mean (95% CI) was 1.16 g/dL (0.98, 1.34) for the EDS group and 1.29 g/dL (1.10, 1.47) for the QW group. The difference between dosing groups for mean [95% CI] change in hemoglobin level from baseline to Week 13 was similar, whether determined using the LVCF (-0.04 g/dL [-0.26, 0.17]) or the available-data (0.13 g/dL [-0.11, 0.36]) method (Table 2). Calculated by either method, the upper limit of the 95% CI was <0.75 g/dL, supporting the noninferiority of EDS compared with QW dosing. Analysis of individual strata with large sample sizes, such as screening hemoglobin <or ≥10 g/dL for each dosing schedule (QW, Q2W, or Q3W), showed a similar lack of differences between treatment groups.

**Table 2:** Change in hemoglobin from baseline to Week 13

	QW	EDS	Difference
	(n = 374)	(n = 378)	(QW - EDS)
LVCF method			
n^a^	374	375	
Least squares mean (95% CI)	0.90 (0.74, 1.07)	0.95 (0.79, 1.11)	-0.04 (-0.26, 0.17)^b^
Available data method			
n^a,c^	257	253	
Least squares mean (95% CI)	1.29 (1.10, 1.47)	1.16 (0.98, 1.34)	0.13 (-0.11, 0.36) ^b^

^a^Three patients in the EDS group were missing screening hemoglobin and were omitted from the ANOVA model

^b^The least squares means and CIs of the treatment difference were computed from an ANOVA model after adjusting for the stratification factors at randomization (chemotherapy cycle length, screening hemoglobin, and tumor type)

^c^Patients missing hemoglobin values at Week 13 were omitted from the analysis

QW = once weekly; EDS = extended dosing schedule; LVCF = last value carried forward; CI = confidence interval; ANOVA = analysis of variance

**Figure 3:**
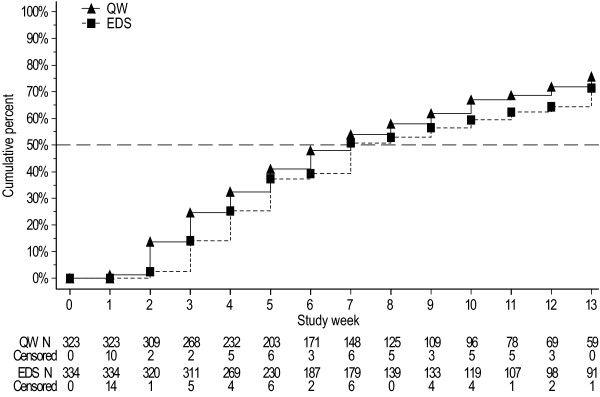
**Kaplan-Meier curve of the time to target hemoglobin from baseline to Week 13**. The time to reach the target hemoglobin of ≥11.0 g/dL was similar for both treatment groups.

The Week 13 KM proportion (95% CI) of patients achieving the target hemoglobin level of ≥11 g/dL was 71% (66, 77) in the EDS group and 76% (70, 81) in the QW group (Table 3). When analyzed by baseline stratification factors, anemic patients (with screening hemoglobin <10 g/dL) with nonmyeloid malignancies excluding lung/gynecologic malignancies who were receiving Q3W chemotherapy in the EDS dosing group (n = 37) achieved the target hemoglobin from baseline to end of treatment in greater proportion (KM% [95% CL]) than those in the QW dosing group (n = 36) (100% [100, 100] vs. 82% [67, 98]). The KM median [95% CI] time in weeks to target hemoglobin was similar between the EDS (7.0 [7.0, 9.0]) and the QW (7.0 [6.0, 8.0]) groups (Figure 3). Patients in both dosing groups maintained similar mean hemoglobin levels after achieving the target hemoglobin level (Table 3). Eighty-four percent of patients in the EDS group and 74% of patients in the QW group were able to maintain hemoglobin levels between 11 and 13 g/dL. The proportion of patients who exceeded the hemoglobin threshold (≥13.0 g/dL) was lower in the EDS group than the QW group (19% vs. 22%, respectively) from baseline to Week 13 and also from baseline to the end of treatment (31% vs. 37%). Fewer patients experienced a rapid rise in hemoglobin (>2.0 g/dL in 4 weeks for patients receiving darbepoetin alfa QW or Q2W or >1.5 g/dL in 3 weeks for patients receiving darbepoetin alfa Q3W) in the EDS group than in the QW group from baseline to Week 13 (27% vs. 46%, respectively) and from baseline to end of treatment (36% vs. 56%).

**Table 3:** Secondary efficacy endpoints

	QW	EDS
	(n = 374)	(n = 378)
Patients who achieved Hb ≥11 g/dL from baseline to Week 13		
Patients included in the analysis^a^	323	334
KM% (95% CI)	76 (70, 81)	71 (66, 77)
Average Hb after achieving Hb ≥11 g/dL		
Patients included in the analysis^b^	301	304
Mean (95% CI)	11.6 (11.5, 11.7)	11.8 (11.7, 11.9)
Category - %		
<11 g/dL	22	13
11 to 13 g/dL	74	84
>13 g/dL	4	4
Patients who had a hematopoietic response from baseline to Week 13		
Patients included in the analysis	374	378
KM% (95% CI)	59 (53, 64)	53 (48,59)
Patients who had a hematopoietic response from baseline to end of treatment		
Patients included in the analysis	374	378
KM% (95% CI)	86 (82, 91)	84 (79, 88)
Patients who had an RBC transfusion from baseline to Week 13		
Patients included in the analysis	374	378
KM% (95% CI)	25 (20, 29)	20 (16, 24)
Patients who had an RBC transfusion from baseline to end of treatment		
Patients included in the analysis	374	378
KM% (95% CI)	29 (24, 34)	26 (21, 30)

^a^Patients who had Hb values ≤11 g/dL at baseline were included in the analysis

^b^Patients who achieved Hb ≥11 g/dL or who had Hb values ≥11 g/dL at baseline were included in the analysis

QW = once weekly; EDS = extended dosing schedule; Hb = Hemoglobin; CI = confidence interval; KM = Kaplan-Meier

Hematopoietic response, defined as an increase in hemoglobin ≥2 g/dL from baseline or a hemoglobin value of ≥12.0 g/dL in the absence of any transfusions within the previous 28 days, was assessed from baseline to Week 13. The hematopoietic response was slightly lower in the EDS group than in the QW group (53% vs. 59%) (Table 3). A smaller proportion of EDS patients had a hematopoietic response in the groups with screening hemoglobin <10 g/dL, nonmyeloid malignancies excluding lung/gynecologic malignancies, and QW or Q3W chemotherapy strata than did QW patients (data not shown).

Patients who received QW dosing appeared to have a slightly greater number of transfusions compared with the EDS group. This was true for transfusions received from baseline to study Week 13 or from baseline to the end of treatment (Table 3). The mean (SD) number of RBC units transfused from baseline to Week 13 was 0.5 (1.3) units in the EDS group compared with 0.7 (1.4) units in the QW group, and 0.7 (1.6) units and 0.8 (1.7) units, respectively, from baseline to the end of treatment.

### Patient-reported Outcomes

The change in FACT-F score was measured from baseline to Week 13 as a secondary endpoint using the available-data approach (Table 4). A greater mean [95% CI] change in FACT-F score was seen in the EDS group (2.5 [1.2, 3.7]) compared with the QW group (1.8 [0.5, 3.1]), which resulted in a mean (95% CI) difference between groups (QW - EDS) of -0.7 (-2.3, 0.9). When data were analyzed by the stratification factors used at randomization, an interaction between treatment and hemoglobin category at screening was observed, indicating that the difference in FACT-F scores between dosing groups was not consistent across screening-hemoglobin categories. Similar percentages of patients in both treatment groups experienced a clinically significant ≥3-point increase in FACT-F score from baseline through Week 13 or end of treatment (Table 4).

**Table 4:** Change in FACT-F score

	QW	EDS	Difference
	(n = 374)	(n = 378)	(QW - EDS)
Baseline FACT-F score			
Patients included in the analysis^a^	330	337	
Mean (SD)	29.7 (12.4)	29.9 (12.4)	
Change in FACT-F score from baseline to Week 13			
Patients included in the analysis^b^	256	268	
Least squares mean (95% CI) ^c^	1.8 (0.5, 3.1)	2.5 (1.2, 3.7)	-0.7 (-2.3, 0.9)
≥3-point increase in FACT-F score from baseline			
Patients included in the analysis^a^	330	337	
Through Week 13, n (%)	111 (34)	132 (39)	
Through the end of treatment, n (%)	155 (47)	173 (51)	

^a^Patients missing a valid baseline and post-baseline FACT-F score were omitted from the analysis

^b^Patients missing a valid baseline and Week 13 FACT-F score were omitted from the analysis

^c^The least squares means and CIs were computed from an ANOVA model after adjusting for the stratification factors at randomization (chemotherapy cycle length, screening hemoglobin, and tumor type), and baseline FACT-F score.

FACT-F = Functional Assessment of Cancer Therapy-Fatigue; QW = once weekly; EDS = extended dosing schedule; SD = standard deviation; CI = confidence interval; ANOVA = analysis of variance

### Resource Utilization

The mean [SD] number of most clinic procedures from baseline to Week 13 was lower among patients in the EDS group compared with the QW group, including lab tests/blood draws (9.9 [4.2] vs. 12.5 [4.8]) and injections (4.9 [3.6] vs. 8.9 [5.6]). In patients receiving darbepoetin alfa Q2W in the EDS group, the mean (SD) number of lab tests/blood draws was 10.5 (4.2) and the mean (SD) number of injections was 5.5 (3.8). In patients receiving darbepoetin alfa Q3W in the EDS group, the mean (SD) number of lab tests/blood draws was 9.2 (4.1) and the mean (SD) number of injections was 4.1 (3.0). Similarly, over the first 13 weeks of treatment, the mean (SD) number of activities impacted by clinic visits was reported to be lower by patients receiving EDS dosing compared with the QW group with respect to both paid employment (4.1 [7.8] in the QW group; 2.9 [6.0] in the EDS group; 3.0 [6.0] in patients receiving darbepoetin alfa Q2W in the EDS group; and 2.7 [6.1] in patients receiving darbepoetin alfa Q3W in the EDS group) and house/yard work (6.7 [8.9] in the QW group; 4.9 [7.4] in the EDS group; 4.6 [7.6] in patients receiving darbepoetin alfa Q2W in the EDS group; and 5.4 [7.1] in patients receiving darbepoetin alfa Q3W in the EDS group).

### Safety

Over the course of this study, most patients (95%) experienced at least one adverse event (Table 5). The type and incidence of adverse events were similar between the EDS and QW dosing groups, with fatigue (22% in both groups) being the most common. The only adverse event with a ≥5% difference between groups was anemia, with an incidence of 6% in the EDS group and 11% in the QW group.

**Table 5:** Adverse events reported on study

Type of Adverse Event	QW (n = 374) n (%)	EDS (n = 378) n (%)
Patients who had any adverse events	357 (95)	360 (95)
Patients who had adverse events of historical interest	106 (28)	100 (26)
Neoplasms benign, malignant and unspecified	59 (16)	54 (14)
Embolism/thrombosis	23 (6)	21 (6)
Deep vein thrombosis	14 (4)	9 (2)
Pulmonary embolism	2 (1)	9 (2)
Phlebitis	2 (1)	2 (1)
Jugular vein thrombosis	0 (0)	1 (<1)
Subclavian vein thrombosis	0 (0)	1 (<1)
Superior vena caval occlusion	0 (0)	1 (<1)
Thrombophlebitis superficial	2 (1)	1 (<1)
Thrombosis	2 (1)	1 (<1)
Atrial thrombosis	1 (<1)	0 (0)
Phlebitis superficial	1 (<1)	0 (0)
Arrhythmias	19 (5)	12 (3)
Congestive heart failure	12 (3)	10 (3)
Hypertension	10 (3)	9 (2)
Cerebrovascular accident	2 (1)	7 (2)
Myocardial infarction/coronary artery disorders	4 (1)	5 (1)
Immune system disorders	0 (0)	3 (1)
Seizure	3 (1)	2 (1)
Patients who had treatment-related adverse events	18 (5)	14 (4)
Patients who had serious adverse events	154 (41)	133 (35)
Patients who had serious treatment-related adverse events	2 (1)	3 (1)
Pulmonary embolism	0 (0)	2 (1)
Arthralgia	0 (0)	1 (<1)
Neutropenia	1 (<1)	0 (0)
Myocardial infarction	1 (<1)	0 (0)
Pyrexia	1 (<1)	0 (0)
Patients who had life-threatening adverse events	22 (6)	16 (4)
Patients who discontinued from the study because of adverse events	58 (16)	46 (12)
Patients who died	39 (10)	39 (10)

QW = once weekly; EDS = extended dosing schedule

The incidence of serious adverse events was slightly lower in the EDS group (133 patients, 35%) than in the QW group (154 patients, 41%). Serious adverse events occurring in ≥5% of patients in either the EDS or QW treatment group were disease progression (5% each), dehydration (6%, 5%), and pneumonia (2%, 5%). Treatment-related adverse events were reported in 14 EDS patients (4%) and 18 QW patients (5%). In 3 EDS patients (1%) and 2 QW patients (1%), these treatment-related adverse events were considered serious (Table 5). One patient in the QW dosing group experienced a moderate myocardial infarction at Week 22, which resolved after 7 days. A second QW patient experienced severe neutropenia and severe pyrexia during the first study week that resolved after 4 days. An EDS patient experienced a life-threatening pulmonary embolism at Week 18 that resolved after 15 days. A second EDS patient experienced severe arthralgia during the first study week that resolved after 6 days. A third EDS patient experienced a severe pulmonary embolism at Week 2 that resolved after 4 days. All 5 patients continued on study. Life-threatening adverse events were reported for 16 EDS patients (4%) and 22 QW patients (6%). Overall, 78 patients (n = 39 [10%] in each group) died on study. The only adverse event leading to death in >1% of patients (n = 11 [3%] in each group) was disease progression, which was also the most common adverse event leading to study discontinuation (4% in each group). During the course of this study, 46 patients (12%) in the EDS group, and 58 patients (16%) in the QW group discontinued from the study because of adverse events.

The incidence of thromboembolic events was similar between dosing groups, occurring in 6% of patients in each group (Table 5). This rate was consistent with that described in the prescribing information for darbepoetin alfa. In both EDS and QW groups, deep vein thrombosis (2% EDS, 4% QW), pulmonary embolism (2% EDS, 1% QW), and phlebitis (1% in each group) were the most common thromboembolic events. Among patients who experienced thromboembolic events, 11 patients (52%) in the EDS group and 15 patients (65%) in the QW group had Stage IV disease.

A separate analysis of adverse events due to safety concerns of historic interest was undertaken. These included: neoplasms benign, malignant, and unspecified (including cysts and polyps); embolism/thrombosis; arrhythmias; congestive heart failure; hypertension; cerebrovascular accident; myocardial infarction/coronary artery disorders; immune system disorder; and seizure. One hundred EDS patients (26%) and 106 QW patients (28%) reported such events. Neoplasms benign, malignant, and unspecified was the most frequent category, occurring in 54 EDS patients (14%) and 59 QW patients (16%) (Table 5).

Neutralizing antibodies to darbepoetin alfa were not detected in any patient (n = 746 patients with samples available for testing). Binding, nonneutralizing antibodies to darbepoetin alfa were observed in 56 (7.5%) of the 746 patients who had test results. This included pre-existing antibodies in 49 (6.6%) patients who were antibody-positive at screening and 7 (0.9%) patients who were antibody-positive only at post-treatment. These 7 patients all developed a low-level antibody response (< 0.5 μg/mL).

## Discussion

In this study of patients diagnosed with nonmyeloid malignancies receiving multicycle chemotherapy and treated with darbepoetin alfa for CIA, the primary objective was to establish the noninferiority of an EDS regimen compared with a QW dosing regimen. The EDS and QW dosing groups in the study achieved a similar mean change in hemoglobin from baseline to Week 13 (1.16 g/dL and 1.29 g/dL, respectively, using the available data approach, or 0.95 and 0.90 g/dL, respectively, using the LVCF approach). We observed that the upper limit of the 95% CI for the difference between groups was less than 0.75 g/dL, thus supporting the noninferiority of EDS dosing compared with QW dosing regimens. In addition, the number of patients who underwent transfusions was similar in both groups at 20% EDS and 25% QW from baseline to Week 13, and 26% EDS and 29% QW from baseline to end of treatment. Both EDS and QW dosing schedules were well tolerated, with similar safety profiles. These results suggest that darbepoetin alfa can be administered QW, Q2W (an unindicated schedule), or Q3W for the treatment of CIA in patients with nonmyeloid malignancies, allowing synchronization with a variety of chemotherapy regimens.

These findings are aligned with previous studies that have compared EDS regimens of darbepoetin alfa to QW regimens and have shown equivalent efficacy. One study of darbepoetin alfa 200 μg Q2W vs. Epoetin alfa QW in 1209 patients with CIA targeted hemoglobin levels of 11 to 13 g/dL and demonstrated noninferiority of the Q2W regimen [10]. In a study of patients receiving darbepoetin alfa 500 μg Q3W vs. 2.25 μg/kg QW by Canon et al [11], a comparable number in each group achieved a target hemoglobin range of 11 to 13 g/dL as did patients in the current study. Additionally, these authors also reported that the Q3W arm was noninferior to the QW arm in regards to incidence of RBC transfusion from Week 5 to end of study (23% in the Q3W group vs. 30% in the QW group), which is consistent with our observations.

We also evaluated the impact of EDS and QW administrations of darbepoetin alfa on patient-reported outcomes and resource utilization. Although no major differences were observed between dosing groups in the mean change in FACT-F scores from baseline to Week 13, overall improvements in FACT-F scores compared favorably with other studies of EDS vs. QW dosing, with approximately 35% of patients achieving a 3-point improvement by Week 13 and about 50% by end of treatment [10-13]. In terms of resource utilization, the number of many types of clinic procedures and the number of activities impacted by clinic visits (such as paid employment and house/yard work) were lower in the EDS group compared with the QW group; this observation was also seen when the separate Q2W and Q3W dosing groups in the EDS arm were compared with the QW group.

Thromboembolic events represent a recognized risk that is reflected in product labeling for ESAs as a class. Rates of embolism/thrombosis reported in this study were similar to those previously reported [6]. Other darbepoetin alfa investigations examining EDS dosing have reported thromboembolic rates similar to that observed in QW dosing, supporting the safety of this dosing regimen in CIA [10-12]. Although the safety profile was similar for both dosing groups, this study was unable to provide comparative safety data for darbepoetin alfa because of the lack of an untreated control group.

A large proportion of patients did not complete the study (39% EDS, 47% QW). Administrative/investigator decisions were responsible for the most study discontinuations; the most frequent reasons given were cessation of chemotherapy and lack of response to the study dose of darbepoetin alfa. In spite of the high attrition rate, the reasons for study discontinuation were balanced between groups and the resulting analysis set was representative of the study population.

While the use of ESAs for the effective treatment of CIA is well supported by the evidence base, data recently published from eight individual studies in patients receiving ESAs for cancer-related anemia have raised concerns regarding increased mortality [3,14-20]. These studies explored experimental, unindicated ESA use either at high hemoglobin targets or in non-CIA indications. Product labeling for ESAs has been revised to state that ESAs shortened overall survival and/or time to tumor progression in clinical studies in patients with breast, non-small cell lung, head and neck, lymphoid, and cervical cancers [6]. Legitimate concerns remain that these risks have not been adequately excluded in the labeled setting and have prompted initiation of additional large, well-controlled studies specifically exploring the effect of ESAs on mortality and tumor progression when administered in accordance with approved labeling. The current study did not demonstrate any apparent difference in survival or progression between the different darbepoetin alfa regimens, but was not powered to address questions of survival or tumor progression and had no untreated concurrent control group for comparison.

This study provides evidence for the utility and efficacy of darbepoetin alfa extended dosing, and for the ability to synchronize dosing with chemotherapy administration. This extended dosing offers potential advantages for patient convenience and clinic efficiency.

## Conclusion

Patients receive chemotherapy on a variety of schedules (QW, Q2W, Q3W or Q4W). The ability to synchronize therapy for CIA with chemotherapy could allow for greater patient convenience and may enhance compliance - provided efficacy is comparable. In this study, we demonstrated the noninferiority of darbepoetin alfa given on an EDS vs. QW dosing regimen, with similar mean changes in hemoglobin, proportions of patients receiving transfusions, and safety profiles. Patients receiving darbepoetin alfa on the EDS schedule had fewer activities of daily living impacted by clinic visits. Thus, EDS darbepoetin alfa offers comparable efficacy with QW dosing but with potential enhanced benefits for patient quality of life.

## Competing interests

Lee Schwartzberg is a consultant and a member of the Speakers' Bureau for Amgen Inc. Ronald Burkes has consulted for Amgen Inc., Roche, and AstraZeneca, has received honoraria from Amgen Inc., Roche, Sanofi-Aventis, Eli Lilly, and AstraZeneca; and has been a member of advisory committees and Speakers' Bureaus for Amgen Inc., Roche, Sanofi-Aventis, Eli Lilly, and AstraZeneca. Barry Mirtsching received honoraria and funding from Amgen Inc. (research payments related to study performance). Timothy Rearden received funding, is a member of an advisory committee, and on the Speakers' Bureau for Amgen Inc. Peter Silberstein is a member of the the Speakers' Bureau for Amgen Inc. Lorrin Yee received funding from Amgen Inc. Amy Inamoto and Tom Lillie are both employees and shareholders of Amgen Inc.

## Authors' contributions

All authors assisted in analyzing and interpreting the data, as well as drafting and approving the final manuscript. LS and TL were involved in study conception and design. LS, RB, BM, TR, PS, and LY were involved in collecting data.

## Pre-publication history

The pre-publication history for this paper can be accessed here:

http://www.biomedcentral.com/1471-2407/10/581/prepub
